# Rivaroxaban – an oral, direct Factor Xa inhibitor – lessons from a broad clinical study programme

**DOI:** 10.1111/j.1600-0609.2009.01230.x

**Published:** 2009-05

**Authors:** Sylvia Haas

**Affiliations:** Institut für Experimentelle Onkologie und Therapieforschung, Technische Universität MünchenMünchen, Germany

**Keywords:** anticoagulation, atrial fibrillation, Factor Xa inhibitor, rivaroxaban, venous thromboembolism

## Abstract

Anticoagulants are recommended for the prevention and treatment of venous thromboembolism (VTE), prevention of stroke in patients with atrial fibrillation (AF) and secondary prevention in patients with acute coronary syndrome (ACS). There is a clinical need for novel anticoagulants offering improvements over current standard of care, such as fixed oral dosing and no need for routine monitoring. Rivaroxaban, an oral, once-daily, direct Factor Xa inhibitor, has recently completed the RECORD phase III programme for the prevention of VTE in patients undergoing total hip or knee replacement (THR or TKR), an indication for which it is approved in Europe and Canada. It is being investigated in large-scale phase III studies for VTE treatment and prevention of stroke in patients with AF, and phase III studies will soon commence for secondary prevention in patients with ACS. Phase I studies demonstrated that no routine anticoagulation monitoring was required, while phase II studies suggested that fixed daily doses had a wide therapeutic window. The four RECORD studies consistently showed that rivaroxaban was significantly more effective than enoxaparin in the prevention of VTE after THR and TKR, with a similar safety profile. This review describes the development of this novel anticoagulant, from bench to bedside.

Anticoagulants are recommended for a broad spectrum of indications, including the prevention and treatment of venous thromboembolism (VTE; comprising deep vein thrombosis [DVT] and pulmonary embolism [PE]) (1, 2), the prevention of stroke in patients with atrial fibrillation (AF) (3) and secondary prevention in patients with acute coronary syndrome (ACS) (4).

Without prophylaxis, DVT occurs in 10–40% of general surgical or medical patients (1). Patients undergoing major orthopedic surgery are at a higher risk; without prophylaxis, 40–60% of these patients develop DVT (1). Guidelines for VTE prevention recommend the routine use of thromboprophylaxis with low molecular weight heparins (LMWHs), fondaparinux or vitamin K antagonists (VKAs) for patients undergoing major orthopedic surgery; however, the oral VKAs are rarely used for this indication in Europe (1, 5). ACCP guidelines currently recommend that thromboprophylaxis be continued for at least 10 d, and up to 35 d after total knee replacement (TKR) and total hip replacement (THR) (1). LMWHs and fondaparinux are effective; however, their long-term use is limited by their parenteral route of administration.

Atrial fibrillation is the most common significant cardiac arrhythmia; it predisposes patients to the development of atrial thrombi and is associated with a four to fivefold increase in the risk of stroke as a result of cardioembolism (6–9). Anticoagulants are recommended in patients with these conditions and, due to the nature of these conditions, long-term therapy is required (9). ACS comprises three cardiac diseases: unstable angina, non-ST-elevated myocardial infarction, and ST-elevated myocardial infarction. The underlying cause of ACS is plaque rupture followed by thrombosis in the coronary arteries and, because many patients remain at high risk of recurrent events, they therefore require secondary preventative therapy (10). Therefore, an oral anticoagulant would be advantageous in these indications. VKAs are the only licensed oral anticoagulants and, although they are effective, they have unpredictable pharmacokinetics (PK) and pharmacodynamics (PD), which are affected by drug and food interactions. As a result, VKAs require frequent monitoring and dose adjustment to ensure that their anticoagulant effects remain within the therapeutic range.

Advances in the understanding of the coagulation pathway have enabled the development of novel anticoagulants targeting specific enzymes within the coagulation cascade, including Factor Xa (FXa) and Factor IIa. FXa has been identified as a particularly attractive target for effective anticoagulation: by catalyzing the conversion of prothrombin to thrombin through the prothrombinase complex, one molecule of FXa results in the generation of more than 1000 thrombin molecules (11). Therefore, inhibition of FXa activity may block the amplification of thrombin generation, limiting thrombin-mediated activation of coagulation and platelets, without affecting existing thrombin levels.

Several FXa inhibitors, such as rivaroxaban, apixaban, betrixaban and edoxaban, are currently at advanced stages of development. Rivaroxaban (Bayer Healthcare AG, Wuppertal, Germany) is a novel, oral, direct FXa inhibitor in advanced development for the prevention and treatment of thromboembolic disorders. Rivaroxaban once-daily (od) has recently received approval in the European Union and in Canada for the prevention of VTE in patients undergoing elective total hip or knee replacement (THR or TKR) surgery. This article will review the results of the clinical studies performed to date and summarize the lessons obtained from this broad development programme.

## Clinical pharmacology

Rivaroxaban exhibits predictable, dose-proportional PK, with high oral bioavailability and a rapid onset of action (maximum plasma concentrations are reached after 1.5–2.0 h) (12). Elimination of rivaroxaban from plasma occurs with terminal half-lives of 5–9 h in young individuals, and with terminal half-lives of 12–13 h in subjects aged >75 yrs (13–15). Pharmacodynamic activity correlates closely with plasma concentrations (12).

Rivaroxaban is distributed heterogeneously to tissues and organs, and exhibits only moderate tissue affinity; importantly, it does not substantially penetrate the blood–brain barrier (16). The drug has a dual mode of elimination: two-thirds are metabolized by the liver (mostly via CYP3A4 and CYP2J2), with no major or active circulating metabolites identified, and one-third is excreted unchanged by the kidneys (17–19).

Results of phase I studies showed that body weight, age, and gender did not have a clinically relevant effect on the PK and PD of rivaroxaban (14, 15, 20); therefore, it is likely that fixed doses of rivaroxaban can be administered to patients, irrespective of their weight, age, or gender. This was supported by the phase II studies investigating rivaroxaban for the prevention and treatment of VTE and the PK and PD analyses of these studies (21–28). Furthermore, phase III studies are investigating fixed doses of rivaroxaban. Rivaroxaban demonstrated a low propensity for drug–drug interactions; results of interaction studies have shown no clinically relevant interaction between rivaroxaban and potential concomitant medications in patients receiving anticoagulants for the prevention or treatment of thromboembolic disorders, i.e. naproxen (29), acetylsalicylic acid (30), clopidogrel (31), or digoxin (32). Furthermore, there are no reported food–drug interactions and dietary restrictions are not necessary in patients receiving rivaroxaban (33). Because rivaroxaban has predictable PK and PD and a low propensity for drug interactions, it is unlikely to require monitoring.

As a result of inhibiting FXa, rivaroxaban inhibits thrombin generation (34), thereby preventing clot formation (35). This mechanism of action results in the dose-dependent prolongation of global clotting tests, such as prothrombin time (PT), activated partial thromboplastin time, and HepTest, with rivaroxaban (12, 13). Furthermore, there was a linear correlation between rivaroxaban plasma concentration and PT measured using Neoplastin® (13, 36), suggesting that PT may be used to assess rivaroxaban exposure, if this was necessary.

While there is no specific antidote to reverse the effects of rivaroxaban, *in vitro* and *in vivo* studies suggest that recombinant Factor VIIa (rFVIIa; NovoSeven®) and activated prothrombin complex concentrate (FEIBA®) may reverse the effects of high-dose rivaroxaban (37–39). If strategies such as delaying the next dose of rivaroxaban or discontinuation, mechanical compression, surgical intervention, fluid replacement and haemodynamic support, blood product, or component transfusion fail to control bleeding, administration of rFVIIa or FEIBA may be considered. However, it is important to note that there is currently no experience with the use of these agents in patients receiving rivaroxaban, and re-dosing of these procoagulants should be considered depending on improvement of the patient’s bleeding status.

## Prevention of VTE in patients undergoing elective THR and TKR surgery

### Phase II studies

The efficacy and safety of rivaroxaban for the prevention of VTE in patients undergoing elective THR and TKR surgery were evaluated in four phase II studies involving 2907 patients (23–25, 28). Both od and twice-daily (bid) dosing regimens were investigated in these studies. A similar study design was utilized for each study, including the same assessment parameters and endpoints, enabling comparison of the findings across the different studies. All events were assessed centrally by the same blinded adjudication committees. All venograms were evaluated by the Gothenburg Center, Sweden.

Mandatory, standardized, bilateral venography was carried out 5–9 d after surgery in the open-label study and in the studies investigating bid administration of rivaroxaban, or 6–10 d after surgery in the od study, or earlier if symptomatic. The primary efficacy endpoint in each study was the composite of any DVT (proximal or distal), non-fatal, objectively confirmed PE, and all-cause mortality. The secondary efficacy endpoints included major VTE (composite of proximal DVT, non-fatal, symptomatic, objectively confirmed PE, and VTE-related death). The primary safety endpoint was major bleeding, defined as fatal bleeding, bleeding into a critical organ (retroperitoneal, intracranial, intraocular, or intraspinal), bleeding leading to re-operation, bleeding warranting treatment cessation, clinically overt bleeding leading to a ≥2 g/dL drop in hemoglobin, or bleeding leading to a transfusion of ≥2 units of blood.

### Open-label study – THR

This proof-of-principle, open-label, dose-escalation study was designed to investigate the efficacy and safety of rivaroxaban, relative to enoxaparin, for VTE prevention in patients undergoing THR (25). A total of 641 patients were randomized to receive oral rivaroxaban (2.5–30 mg bid, or 30 mg od) or subcutaneous enoxaparin (40 mg od); rivaroxaban was initiated 6–8 h after surgery and then every 12 h (bid regimens) or 24 h (od regimen). Enoxaparin was first administered the evening before surgery and od thereafter, according to standard European practice. Administration of study drug was continued for 5–9 d after surgery.

The primary efficacy endpoint occurred with similar frequency for rivaroxaban and enoxaparin. There was a flat dose–response relationship between rivaroxaban and the primary endpoint. For the secondary efficacy endpoint (major VTE), the dose–response relationship with rivaroxaban was significant (*P*=0.0108), with increasing doses resulting in a reduced incidence of major VTE. With respect to safety, major bleeding increased dose dependently with rivaroxaban and the dose–response relationship was significant (*P*=0.0008).

The results from this study demonstrated proof of principle for rivaroxaban for the prevention of VTE in patients undergoing elective THR and TKR surgery, supporting its continued assessment in double-blind studies. These findings also provided the first evidence of the feasibility of od dosing with rivaroxaban.

### Twice-daily dosing studies – THR or TKR

Following the proof-of-principle study, two separate double-blind, double-dummy, dose-ranging studies were conducted to assess the efficacy and safety of bid administration of a 12-fold range of rivaroxaban doses (2.5, 5, 10, 20, or 30 mg bid) relative to enoxaparin (24, 28). A total of 722 patients undergoing THR were randomized into one study in Europe (24), and 621 patients undergoing TKR were randomized into a separate study in North America (28). Rivaroxaban was initiated 6–8 h after surgery and continued for 5–9 d. Enoxaparin was administered according to European or North American prescribing information: in the European hip study, patients received enoxaparin 40 mg od, with the first dose given the evening before surgery; in the North American knee study, enoxaparin 30 mg bid was administered every 12 h, with the first dose given the morning after surgery.

There was a flat dose–response relationship for rivaroxaban with respect to the primary efficacy endpoint in the hip (*P*=0.93) and knee studies (*P*=0.29). In the hip study, the incidence of the primary endpoint ranged between 6.9% and 18.2% for rivaroxaban, which was similar to that observed with enoxaparin (17%). The incidence of the primary efficacy endpoint was also similar between rivaroxaban and enoxaparin in the knee study (23.3–40.4% for rivaroxaban and 44.3% for enoxaparin). Differences in the incidence of the primary endpoint between the hip and knee studies are in line with previous studies showing a higher rate of venographically detected DVT in TKR compared with THR (40). The incidence of major VTE was similar for all rivaroxaban doses and enoxaparin in both studies. For the primary efficacy endpoint, a flat dose–response relationship was observed between rivaroxaban and major VTE. The safety profile of rivaroxaban was consistent across the two studies. For rivaroxaban total daily doses 5–20 mg, the incidence of major bleeding events was similar to enoxaparin. As expected, a dose–response relationship was observed for major bleeding events (*P*=0.045 and *P*=0.0007 in the hip and knee studies, respectively).

The findings from these two studies suggested that rivaroxaban has a wide therapeutic window, which may indicate a favorable risk–benefit profile. Balancing efficacy and bleeding risk, it was concluded from these studies that the optimal dose range was a total daily dose of 5–20 mg.

### Pooled analysis

The two double-blind studies were designed to allow the results to be pooled and analyzed based on a larger population size, and to determine if any differences in the results existed between the efficacy and safety of rivaroxaban in patients undergoing elective THR or TKR. The results of this study demonstrated that there were no significant differences between the dose–response relationships with rivaroxaban after THR or TKR: there was no significant dose trend for the primary efficacy endpoint (*P*=0.39), whereas there was a significant dose trend for the primary safety endpoint (*P*<0.001) (41). This study also confirmed that, based on both efficacy and safety, a total daily dose of 5–20 mg was the optimal dose range.

### Once-daily dosing study – THR

Earlier studies showed that rivaroxaban inhibited FXa activity for around 12 h after administration (13); therefore, bid administration was investigated initially. However, it was subsequently found that thrombin generation remained inhibited for 24 h after rivaroxaban administration, suggesting that od administration would be possible (34). This was supported by the efficacy and safety of the 30 mg od dose in the open-label study. Therefore, this study was conducted to determine the efficacy and safety of od dosing of rivaroxaban across an eightfold dose range (5–40 mg) (23). Rivaroxaban was administered 6–8 h after surgery and then every 24 h. Enoxaparin was first administered the evening before surgery, and od thereafter. Administration was continued for 5–9 d after surgery for rivaroxaban and enoxaparin.

The incidence of the primary efficacy endpoint was similar between rivaroxaban (across the eightfold dose range) and enoxaparin. Although there was a tendency towards a lower incidence of the primary efficacy endpoint with increasing doses of rivaroxaban, the trend was not significant (*P*=0.0852). These results were similar to those obtained in the bid studies (24), indicating similar efficacy with od dosing of rivaroxaban. The observed incidence of major VTE was lower in all rivaroxaban dose groups compared with enoxaparin, except for the rivaroxaban 5 mg dose group. A dose–response relationship was observed between rivaroxaban and major VTE (*P*=0.0072). For the primary safety endpoint, the two lower doses of rivaroxaban (5 and 10 mg) showed a similarly low rate of major bleeding compared with enoxaparin (2.3% and 0.7%, respectively, relative to 1.9%). There was a significant trend in the dose–response relationship between rivaroxaban and major bleeding (*P*=0.039). Safety endpoints were similar to those observed in the bid studies (24).

**Figure 1:**
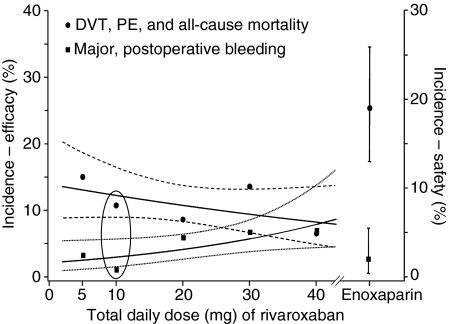
Dose–response relationships between rivaroxaban and the primary efficacy endpoint and the primary safety endpoint in the od study investigating rivaroxaban for the prevention of VTE after major orthopedic surgery (23).

When both efficacy and safety were considered, it was concluded that the optimum dose of rivaroxaban for VTE prevention was 10 mg od, a dose within the range identified in the bid studies (Fig. 1) (23).

### Adverse events and laboratory parameters

In all of the studies, the incidence of serious treatment-emergent adverse events considered to be drug-related was similar for rivaroxaban and enoxaparin, and no dose arm was stopped because of safety concerns. There was no evidence of compromised liver function attributable to rivaroxaban in these studies, and the results of the liver function tests were similar to those obtained with enoxaparin. The incidence of liver enzyme elevations was not dose-related for rivaroxaban, and any elevations observed were transient (23–25, 28).

### PK and PD analyses

Rivaroxaban demonstrated predictable PK and PD in patients undergoing major orthopedic surgery (27). Age, body weight, and renal function had a moderate effect on rivaroxaban exposure; however, the effect was not considered clinically relevant (27). Furthermore, a subgroup analysis of the two bid studies and the dose-escalation study showed that the dose–response relationships with rivaroxaban for efficacy and safety were not affected by age, gender or weight (42).

As a result, phase III studies investigating rivaroxaban for the prevention of VTE in patients undergoing major orthopedic surgery have enrolled patients with no upper age limit and those with mild or moderate hepatic impairment. Furthermore, fixed doses of rivaroxaban are being investigated, irrespective of body weight, age or gender.

### The phase III RECORD program

Based on the results from the phase II studies, rivaroxaban 10 mg od was selected to be investigated in the phase III RECORD programme, comprising four large studies in more than 12 500 patients undergoing elective THR or TKR.

In all of the studies, the primary efficacy endpoint was the composite of DVT, non-fatal PE and all-cause mortality, and the main secondary efficacy endpoint was major VTE (the composite of proximal DVT, non-fatal PE, and all-cause mortality). The primary safety endpoint was major bleeding (Table 1).

**Table 1:** Incidence of venous thromboembolism and bleeding events across the four RECORD studies (43–46)

	RECORD1 (THR)		RECORD2 (THR)		RECORD3 (TKR)		RECORD4 (TKR)	
Enoxaparin, 40 mg od	Rivaroxaban, 10 mg od	Enoxaparin, 40 mg od	Rivaroxaban, 10 mg od	Enoxaparin, 40 mg od	Rivaroxaban, 10 mg od	Enoxaparin, 30 mg bid	Rivaroxaban, 10 mg od
Endpoint	5 wks		10–14 d	5 wks	10–14 d		10–14 d	
Efficacy endpoints								
Total VTE (primary endpoint)								
% (*n*)	3.7 (58/1558)	1.1 (18/1595)	9.3 (81/869)	2.0 17/864)	18.9 (166/878)	9.6 (79/824)	10.1 (97/959)	6.9 (67/965)
*P*-value	<0.001		<0.0001		<0.001		0.012	
Major VTE								
% (*n*)	2.0 (33/1678)	0.2 (4/1686)	5.1 (49/962)	0.6 (6/961)	2.6 (24/925)	1.0 (9/908)	2.0 (22/1112)	1.2 (13/1122)
*P*-value	<0.001		<0.0001		0.01		0.124	
Symptomatic VTE								
% (*n*)	0.5 (11/2206)	0.3 (6/2193)	1.2 (15/1207)	0.2 (3/1212)	2.0 (24/1217)	0.7 (8/1201)	1.2 (18/1508)	0.7 (11/1526)
*P*-value	0.22		0.0040		0.005		0.187	
Bleeding endpoints, % (*n*)								
Major bleeding	0.1 (2/2224)	0.3 (6/2209)	<0.1 (1/1229)	<0.1 (1/1228)	0.5 (6/1239)	0.6 (7/1220)	0.3 (4/1508)	0.7 (10/1526)
Clinically relevant non-major bleeding	2.4 (54/2224)	2.9 (65/2209)	2.7 (33/1229)	3.3 (40/1228)	2.3 (28/1239)	2.7 (33/1220)	NA^1^	NA^1^

All *P*-values for efficacy calculated from absolute risk reduction.^1^Correction added 16 March 2009 after online publication. Previous values have been replaced by 'NA'

od, once daily; RRR, relative risk reduction; THR, total hip replacement; TKR, total knee replacement; VTE, venous thromboembolism; NA, Not Applicable.

Current guidelines recommend extended prophylaxis for patients undergoing THR; however, these recommendations have not been implemented into clinical practice in many countries. Therefore, RECORD2 investigated the efficacy and safety of extended thromboprophylaxis with rivaroxaban (5 wks) compared with short-term enoxaparin (10–14 d) in patients undergoing THR (43). The results of this study demonstrated that extended prophylaxis with rivaroxaban 10 mg od was superior to short-term prophylaxis with enoxaparin 40 mg od for the prevention of VTE, including symptomatic events, after THR (Table 1) (43). Despite rivaroxaban being given for 3 wks longer than enoxaparin, the incidence of major bleeding at 5 wks was 0.1% in both groups. This study confirmed the benefits of extended prophylaxis over short-term prophylaxis and the safety of its use.

The RECORD1 and 3 studies were designed to compare rivaroxaban 10 mg od (starting 6–8 h after surgery) with enoxaparin 40 mg od (starting the evening before surgery) given for 31–39 d (extended prophylaxis) after THR (RECORD1) (44) and 10–14 d (short-term prophylaxis) after TKR (RECORD3) (45). In both studies, rivaroxaban was significantly more effective than enoxaparin for the prevention of VTE (Table 1) (44, 45). RECORD3 also showed a significant reduction in symptomatic VTE, and whereas RECORD1 showed a general trend for reduction in symptomatic VTE, this was not significant.

RECORD4 compared the efficacy and safety of oral rivaroxaban 10 mg od with the North American regimen of enoxaparin 30 mg bid, given subcutaneously (10–14 d) in patients undergoing TKR (46). Rivaroxaban was significantly superior to enoxaparin for the primary efficacy endpoint, with no significant difference in the rates of major bleeding between the two groups (Table 1).

There was no evidence of compromised liver function attributable to rivaroxaban in all four studies. The incidence rates of predefined abnormal liver function tests (alanine aminotransferase [ALT] levels elevated to three times the upper limit of normal [ULN] and bilirubin greater than twice the ULN) were similar in the rivaroxaban and enoxaparin groups (43–46).

Rivaroxaban head-to-head comparison with enoxaparin in these four studies showed the efficacy and safety of rivaroxaban in the prevention of VTE in patients undergoing major orthopedic surgery. The superiority of rivaroxaban for the primary efficacy endpoint was demonstrated in all four studies. Rivaroxaban also showed a good safety profile, with low incidence of major bleeding similar to that observed with enoxaparin, and no evidence of drug-induced liver injury.

## Prevention of VTE in medically ill patients

A phase III study has also been initiated to investigate the efficacy and safety of prophylaxis with rivaroxaban 10 mg od (for up to 5 wks), compared with short-term enoxaparin, in hospitalized, medically ill patients (http://www.clinicaltrials.gov; NCT00571649).

## Treatment of VTE

### Phase II studies

The efficacy and safety of rivaroxaban for the treatment of VTE were assessed in two phase IIb studies: ODIXa-DVT (21) and EINSTEIN-DVT (22) (Table 2). In both studies, patients with acute, symptomatic, objectively confirmed, proximal DVT without symptomatic PE received double-blind rivaroxaban or open-label standard therapy (LMWH/heparin and a VKA) for 3 months.

**Table 2:** Efficacy and safety results of the ODIXa-DVT and EINSTEIN-DVT studies (21, 22)

	Rivaroxaban				
ODIXa-DVT study	10 mg bid (*n* = 100)	20 mg bid (*n* = 98)	30 mg bid (*n* = 109)	40 mg od (*n* = 112)	Enoxaparin + VKA (*n* = 109)
Improvement in thrombus burden without recurrent VTE at 3 wks, %	53.0	59.2	56.9	43.8	45.9
Recurrent DVT, PE, and VTE-related death at 3 months, *n* (%)	2 (1.9)	2 (2.0)	2 (1.8)	3 (2.6)	1 (0.9)
Major bleeding, *n* (%)	2 (1.7)	2 (1.7)	4 (3.3)	2 (1.7)	0 (0.0)
	Rivaroxaban			
EINSTEIN-DVT study	20 mg od (*n* = 115)	30 mg od (*n* = 112)	40 mg od (*n* = 121)	LMWH/heparin + VKA (*n* = 101)
Recurrent VTE and thrombus deterioration at 3 months, *n* (%)	7 (6.1)	6 (5.4)	8 (6.6)	10 (9.9)
Major bleeding, *n* (%)	1 (0.7)	2 (1.5)	0 (0.0)	2 (1.5)

bid, twice daily; DVT, deep vein thrombosis; LMWH, low molecular weight heparin; od, once daily; PE, pulmonary embolism; VKA, vitamin K antagonist; VTE, venous thromboembolism.

### ODIXa-DVT

In the ODIXa-DVT study, rivaroxaban 10, 20 or 30 mg bid, or 40 mg od doses were assessed relative to standard therapy (i.e. enoxaparin 1 mg/kg bid followed by a VKA) (21). The primary efficacy endpoint was reduced thrombus burden on day 21 (assessed by quantitative compression ultrasonography; ≥4-point improvement in thrombus score) without recurrent VTE or VTE-related death. The primary efficacy endpoint was achieved in 43.8–59.2% of patients receiving rivaroxaban and in 45.9% of patients receiving standard therapy. The incidence of the primary safety endpoint (major bleeding) was 1.7–3.3% in the rivaroxaban groups; there were no events in the standard therapy group. It was concluded that, over a wide range of doses, the oral, direct FXa inhibitor demonstrated good efficacy and safety for the treatment of acute symptomatic DVT. This was the first phase II trial to use quantitative compression ultrasonography to demonstrate reduced thrombosis burden after initial course of therapy with a new anticoagulant.

### EINSTEIN-DVT

In the EINSTEIN-DVT study, rivaroxaban 20, 30 or 40 mg od were assessed relative to standard therapy (22). The primary efficacy endpoint was the composite of symptomatic, recurrent VTE and deterioration of thrombotic burden, as assessed by compression ultrasound and perfusion lung scan, at 3 months.

The primary efficacy endpoint occurred in 5.4–6.6% of patients receiving rivaroxaban vs. 9.9% in the standard therapy group. The primary safety endpoint (any clinically relevant bleeding) occurred in 2.9–7.5% of patients receiving rivaroxaban vs. 8.8% in the standard therapy group. Major bleeding occurred in 0–1.5% of patients receiving rivaroxaban vs. 1.5% of patients receiving standard therapy.

Overall, the results of this study demonstrated that rivaroxaban 20–40 mg od had good efficacy and safety for the treatment of acute symptomatic DVT. This study, in which deterioration of thrombus burden was a component of the primary endpoint assessment at 3 months, complements the findings of the ODIXa-DVT trial.

### Adverse events

In both studies, there was no evidence of compromised liver function attributable to rivaroxaban during the 3 months of treatment. Increases in liver enzyme levels were not dose dependent for rivaroxaban, and any increases were transient. In the ODIXa-DVT study, rivaroxaban was stopped prematurely in three patients (21). Two patients had early (on the day, or on the day after, initiation of treatment) elevations of ALT and aspartate aminotransferase levels, which returned to levels below the ULN after treatment was stopped. In the third patient, rivaroxaban was stopped after 23 d, when hepatitis B with seroconversion was diagnosed; the patient died of acute liver failure, mostly due to fatal hepatitis B infection, 48 d after starting treatment (21).

### PK and PD analyses

Predictable PK and PD were demonstrated in patients receiving rivaroxaban for the treatment of DVT (27). Furthermore, the PK and PD were similar with od and bid dosing, suggesting that od dosing would not increase either the risk of bleeding or thrombus growth, compared with bid dosing. Demographic factors such as age, renal function and body weight had only moderate effects on the PK and PD, suggesting that fixed doses of rivaroxaban can be administered to patients. This was consistent with findings in both healthy subjects and patients undergoing major orthopedic surgery.

### Phase III dose and regimen selection

In the ODIXa-DVT study, improvement in complete compression ultrasound without recurrent VTE was achieved in 43.8% of patients receiving rivaroxaban 40 mg od and 53.0–59.2% of those receiving the drug bid. These results suggested that the bid regimen could be more effective for thrombus regression at 3 wks (21). At 3 months, the od and bid regimens performed similarly. Therefore, an initial intensified bid regimen (rivaroxaban 15 mg bid for 3 wks) followed by convenient, long-term 20 mg od was selected for investigation in phase III studies.

### The phase III EINSTEIN studies

The efficacy and safety of rivaroxaban for the treatment of VTE are being further assessed in three phase III studies involving approximately 7500 patients – EINSTEIN-DVT, EINSTEIN-PE and EINSTEIN-EXTENSION (http://www.clinicaltrials.gov; NCT00440193, NCT00439777, and NCT00439725).

EINSTEIN-DVT and EINSTEIN-PE are multicenter, randomized, open-label studies. Patients with confirmed symptomatic DVT (for the DVT study) or PE (for the PE study) are randomized to receive either standard therapy (enoxaparin, followed by a VKA) or rivaroxaban. Rivaroxaban is being administered at 15 mg bid for the first 3 wks of treatment, after which patients will receive a dose of 20 mg od for a predefined treatment period of 3, 6 or 12 months.

The EINSTEIN-EXTENSION study is recruiting patients who have been treated for 6 or 12 months with rivaroxaban or a VKA. These patients will be randomized to receive double-blind rivaroxaban 20 mg od or placebo for a further 6 or 12 months.

## Prevention of stroke in patients with AF

The phase II studies investigating the efficacy and safety of rivaroxaban for the treatment of VTE were also used to select 20 mg od for investigation in phase III studies for the prevention of stroke in AF (Table 3).

**Table 3:** The clinical development programme for rivaroxaban

Trial	Indication	Trial design	Notes
Phase III RECORD	VTE prevention in patients undergoing major orthopedic surgery	>11 000 patients Hip replacement or knee replacement vs. standard therapy (enoxaparin)	Approved in EU and Canada; US NDA filed in July 2008
Phase III MAGELLAN	VTE prevention in the medically ill	vs. standard therapy (enoxaparin)	
Phase III ROCKET AF	Prevention of stroke in patients with atrial fibrillation	∼14 000 patients Non-inferiority vs. standard therapy (warfarin)	Regulatory filing expected in 2010
Phase III EINSTEIN	VTE treatment	∼7500 patients vs. standard therapy	Regulatory filing expected in 2010
Phase III ATLAS 2 TIMI 51	Secondary prevention of fatal and non-fatal cardiovascular events in patients with acute coronary syndrome (ACS)	16 000 patients In addition to standard therapy	Regulatory filing expected in 2012

Rivaroxaban 20 mg od is being compared with warfarin for the prevention of stroke in approximately 14 000 patients with AF in the Rivaroxaban Once daily oral direct FXa inhibition Compared with vitamin K antagonism for prevention of stroke and Embolism Trial in Atrial Fibrillation (ROCKET AF) study. Patients with moderate renal impairment (creatinine clearance 30–49 mL/min) will receive a fixed dose of 15 mg od (http://www.clinicaltrials.gov; NCT00403767).

A separate study, the J-ROCKET AF study, is also being conducted in Japan, with rivaroxaban 15 mg od (10 mg od for patients with moderate renal impairment) being compared with warfarin (http://www.clinicaltrials.gov; NCT00494871).

## Secondary prevention in patients with ACS

A phase IIb study investigating the use of rivaroxaban in patients with ACS has recently been completed. The Anti-Xa Therapy to Lower cardiovascular events in Addition to aspirin with/without thienopyridine therapy in Subjects with Acute Coronary Syndrome [ATLAS ACS (TIMI 46)] study assessed safety and efficacy in approximately 3500 patients with recent, non-ST-elevated myocardial infarction, ST-elevated myocardial infarction, or unstable angina. All patients received standard antiplatelet therapy of low-dose ASA, and a thienopyridine (such as clopidogrel) at the physician’s discretion. Patients were then randomized to additionally receive either rivaroxaban or a placebo for six months. Escalating total daily doses of rivaroxaban, ranging from 5 mg up to 20 mg (eight dosing regimens in total), were administered od or bid. Safety was evaluated by measuring clinically significant bleeding, defined as a composite of TIMI major bleeding, TIMI minor bleeding and any reported bleeding event requiring medical attention. As expected, patients in the rivaroxaban regimens had higher rates of bleeding vs. placebo when administered in combination with antiplatelet therapy. However, no study arm was halted due to increased bleeding. No evidence of drug-induced liver injury was seen. Although the study was not powered to demonstrate significance in the composite efficacy endpoint of death, myocardial infarction, stroke or severe recurrent ischemia requiring revascularization, a definite trend towards reduction in cardiovascular events was also observed (47). Two doses of rivaroxaban, 2.5 and 5 mg bid, have been identified that will be investigated in a phase III study, ATLAS 2 TIMI 51. This study is expected to enroll up to 16 000 patients, commencing in December 2008 (Table 3).

## Discussion

Rivaroxaban is a novel, oral, direct FXa inhibitor in advanced clinical development for the prevention and treatment of thromboembolic disorders. The drug has undergone extensive evaluation in phase II studies for the prevention and treatment of VTE. These studies suggested that rivaroxaban had a wide therapeutic window, with similar efficacy and safety to standard therapy.

Rivaroxaban is currently being investigated in large-scale phase III studies in two indications, treatment of VTE and prevention of stroke in patients with AF, with phase III studies to be started soon for another indication, secondary prevention in patients with ACS. Results from all four phase III studies investigating rivaroxaban once daily for prevention of VTE after elective THR and TKR surgery are now available. In RECORD1 and RECORD3, the drug was significantly more effective than enoxaparin for the prevention of VTE in patients undergoing THR and TKR, respectively. In RECORD2, extended prophylaxis with rivaroxaban demonstrated superior efficacy to short-term prophylaxis with enoxaparin in patients undergoing THR. In RECORD4, rivaroxaban was superior to the North American regimen of enoxaparin for the primary efficacy endpoint. Importantly, safety was not compromised: rivaroxaban was not associated with a statistically significant increased risk of major bleeding in all four phase III studies. The RECORD trial programme confirms that direct FXa inhibition can significantly reduce the burden of VTE in high-risk orthopedic patients, and oral administration has the potential to increase the uptake of postdischarge prophylaxis in patients undergoing THR, thereby facilitating the implementation of guidelines in clinical practice.

Although rFVIIa or FEIBA may reverse the effects of high-dose rivaroxaban in case of over-anticoagulation or in patients needing emergency surgery (37–39), there is currently no experience with the use of these agents in patients receiving rivaroxaban, which could constitute a drawback to the use of this drug.

The terminal half-life of rivaroxaban is prolonged in subjects over 75-yr old compared with younger subjects. However, analyses of the pharmacokinetics of rivaroxaban in patients participating in phase II studies demonstrated that age influenced the pharmacokinetics of the drug, but that the effects were minor and within expectations (26, 27).

Although two-thirds of rivaroxaban are metabolized via the cytochrome P (CYP)450 (mostly CYP3A4 and CYP2J2), (19) the risk of observing clinically relevant drug–drug interactions with rivaroxaban through inhibition or induction of CYP1A2, CYP3A4 and other CYP isoforms is considered to be low,(Unpublished results) and only strong CYP3A4 inhibitors, given at doses resulting in high maximum plasma concentrations, have an effect on the metabolism of rivaroxaban and might lead to a decrease in renal clearance (19).

Other oral anticoagulants, both direct thrombin inhibitors (DTIs) and direct FXa inhibitors, are currently at advanced stages of development. Of these new agents, the direct thrombin inhibitors dabigatran and AZD0837, and the direct FXa inhibitors YM150, betrixaban, edoxaban and apixaban are the most promising. Dabigatran has also been approved in the EU and Canada for the prevention of VTE in adult patients who have undergone elective THR or TKR. Dabigatran is also undergoing phase III studies for the treatment of VTE, and for stroke prevention in AF. Among the FXa inhibitors, apixaban is currently undergoing phase III studies for the prevention and treatment of VTE, as well as for stroke prevention in patients with AF.

As well as demonstrating efficacy and safety in the prevention of VTE after elective hip and knee replacement surgery, the drug is administered orally, once daily, and does not require routine anticoagulation monitoring because of its predictable PK/PD profile. As a result, rivaroxaban has the potential to be an attractive alternative to current anticoagulants, providing effective and well-tolerated anticoagulation in a convenient manner, from hospital to home.
